# Design and application of a target capture sequencing of exons and conserved non-coding sequences for the rat

**DOI:** 10.1186/s12864-016-2975-9

**Published:** 2016-08-09

**Authors:** Minako Yoshihara, Daisuke Saito, Tetsuya Sato, Osamu Ohara, Takashi Kuramoto, Mikita Suyama

**Affiliations:** 1 Medical Institute of Bioregulation, Kyushu University, Maidashi 3-1-1, Higashi-ku, Fukuoka, 812-8582 Japan; 2 AMED-CREST, Japan Agency for Medical Research and Development, Fukuoka, 812-8582 Japan; 3 Department of Technology Development, Kazusa DNA Research Institute, Kisarazu, 292-0818 Chiba Japan; 4 Institute of Laboratory Animals, Graduate School of Medicine, Kyoto University, Kyoto, 606-8501 Japan

**Keywords:** Target capture sequencing, Exome, Conserved non-coding sequence, Rat

## Abstract

**Background:**

Target capture sequencing is an efficient approach to directly identify the causative mutations of genetic disorders. To apply this strategy to laboratory rats exhibiting various phenotypes, we developed a novel target capture probe set, TargetEC (target capture for exons and conserved non-coding sequences), which can identify mutations not only in exonic regions but also in conserved non-coding sequences and thus can detect regulatory mutations.

**Results:**

TargetEC covers 1,078,129 regions spanning 146.8 Mb of the genome. We applied TargetEC to four inbred rat strains (WTC/Kyo, WTC-*swh*/Kyo, PVG/Seac, and KFRS4/Kyo) maintained by the National BioResource Project for the Rat in Japan, and successfully identified mutations associated with these phenotypes, including one mutation detected in a conserved non-coding sequence.

**Conclusions:**

The method developed in this study can be used to efficiently identify regulatory mutations, which cannot be detected using conventional exome sequencing, and will help to deepen our understanding of the relationships between regulatory mutations and associated phenotypes.

**Electronic supplementary material:**

The online version of this article (doi:10.1186/s12864-016-2975-9) contains supplementary material, which is available to authorized users.

## Background

Whole exome sequencing (WES) is used to sequence only the exonic portion of the genome, which comprises 1–2 % of the entire genome. Hence, WES reduces the cost associated with the identification of the causative mutations of a certain disease while maintaining the efficiency of mutation detection in protein-coding regions that might substantially affect the phenotype. Since the use of this method to select an entire set of human exons for sequencing using probe hybridization [1] and its first application for the identification of a gene involved in a genetic disease were reported [2], WES has been successfully applied to the identification of responsible genes and their causative mutations in many diseases (for review, see [3]).

Although WES has been mainly applied to human genetics, some studies have investigated WES application to mouse exome [4, 5], which is among the most widely used animal models in basic molecular biology and biomedical science. Rat has also been used extensively as a model for various human diseases and the genome has been sequenced [6]; however, WES analyses of rat exome have not yet been reported because of the lack of a commercially available exome capture kit. It is possible to design a customized capture kit for the desired target sequences in such species. Some applications of exome sequencing have been described for some species for which commercial capture kits are not available. These species include dogs [7, 8], pigs [9], cows/bison [10], chipmunks [11], and non-human primates [12].

In the conventional WES target capture method, all regions except for exons and their neighboring splice sites are eliminated as targets. As such, mutations in most regulatory regions, which often reside in non-exonic regions, are, in principle, hardly detected despite the fact that mutations in these regions have been found to be responsible for certain diseases such as preaxial polydactyly [13] and the Pierre Robin sequence [14] (for review, see [15–18]). Such mutations are often identified through genome-wide association studies (GWASs). For example, among the published reports of 2304 studies (16,831 associations) registered in the GWAS Catalog (October 30, 2015) [19], the reports of 535 studies (2276 associations) stated that significant markers were found in intergenic regions distant from any annotated genes. These may be candidates for disorder- or risk-associated loci that harbor regulatory mutations, which cannot be detected using conventional WES as this technique targets only the exons in protein-coding genes. Because these regulatory regions are functionally important and have been subjected to selective pressure, they tend to be conserved during the course of evolution [20]. Therefore, to ensure that the target capture procedure covers not only exons for protein-coding genes but also possible regions containing regulatory mutations, these regions, namely conserved non-coding sequences (CNSs), should be included in the target. Advances in vertebrate genome sequencing and subsequent comparative genomic analyses have made it possible to decipher such regions under strong selective constraints. Information about these regions can easily be retrieved from the multiple genome sequence alignments provided by the UCSC Genome Browser [21].

One key step in a WES data analysis is the filtering of known single nucleotide variants (SNVs) that are commonly observed in different individuals and hence thought not to be associated with a certain phenotype of interest. At this stage, filtering efficiency depends on the number of known SNVs commonly found in wild-type samples. The most popular data repository for these variants is dbSNP [22]. The number of rat SNVs in dbSNP (Build 146; November 24, 2015) is approximately 5.1 million, whereas 150.5 million and 99.5 million variants are registered for the human and mouse, respectively. This discrepancy has been considered a disadvantage with regard to rat exome analysis in comparison to human and mouse analyses. Fortunately, rapid progress in genome sequencing and the identification of sequence variations in several rat strains have recently been made [23–26]. These resources concerning sequence variations in rat will allow an efficient focus on variants that might be responsible for a certain phenotype.

Here, we describe the development of a novel rat target capture probe set, TargetEC (target capture for exons and CNSs). This probe set covers not only the annotated exons for protein-coding genes but also CNSs, and thus allows the detection of variations in genomic regions important for regulatory functions. To evaluate its performance, we applied the TargetEC to two normal rat strains, WTC/Kyo and PVG/Seac. In addition, as a proof of concept, we also applied this probe set to two mutant rat strains to demonstrate its ability to identify causative mutations for the phenotypes: WTC-*swh*/Kyo, which exhibits a sparse and wavy hair (*swh*) phenotype [27], and KFRS4/Kyo, which exhibits multiple phenotypes such as congenital cataract formation [28] and ear malformation [29]. This study describes a powerful method by which to identify the causative mutations of various phenotypes in rat strains.

## Results

### Design of target capture regions for sequencing

RefSeq [30] and Ensembl [31] were used for gene annotation; these databases contain 18,573 transcripts for 17,168 genes with coverage of 39.0 Mb of the genome, and 30,404 transcripts for 26,689 genes with coverage of 48.2 Mb of the genome, respectively. By merging these two gene annotations, the genomic regions covered by these annotations increased slightly to 50.8 Mb.

**Fig. 1:**
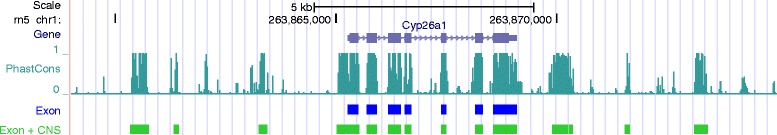
A genome browser view illustrating genome sequence conservation around *Cyp26a1*. The track at the top indicates the structure of *Cyp26a1*, followed by the phastCons conservation score track calculated for the genome alignment of 13 vertebrate species. The last two tracks represent the exons (blue) and exons plus CNSs (green)

Many regions in the genome sequences of vertebrate species exhibit a very high level of conservation, suggesting that selective pressure has acted on these regions to ensure certain functions (Fig. 1). These regions are comprised of not only exons, but also CNSs. A certain fraction of CNSs might correspond to regulatory elements for gene expression, which are not well annotated even in the human and mouse genomes. By including these regions in target capture sequencing, it might be possible to identify regulatory mutations that would not be targets of conventional exome sequencing. In this study, we used phastCons scores [21], which range from 0.0 (low conservation) to 1.0 (high conservation) and were calculated for the genome alignment of 13 vertebrate species (including rat as the reference genome) as an index for genome sequence conservation. We focused only on highly conserved regions with phastCons scores of 1.0, which accounted for 36.1 Mb of the rat genome; of these, 5.4 Mb did not overlap with exons. Here we must note that when designing capture probes, shorter regions must be expanded to at least 100 bp to ensure efficient target region capture. Because non-exonic conserved regions (median: 5 bp) tend to be shorter than exonic regions (median: 132 bp for RefSeq exons), the inclusion of non-exonic conserved regions drastically affects the total lengths of the capture probes. Mainly for this reason, we focused only on regions with phastCons scores of 1.0 for CNS capture. By merging these regions (i.e., exons and CNSs) and expanding the minimum length of the regions to 100 bp, we obtained initial target regions (588,195 regions, total = 137.9 Mb). After optimizing the probe design using the manufacturer’s internal protocols, we finally obtained target regions for TargetEC (1,078,129 regions, total = 146.8 Mb) that were subsequently applied to capture probe design using the SeqCap EZ Developer Library product from Roche NimbleGen.

**Fig. 2:**
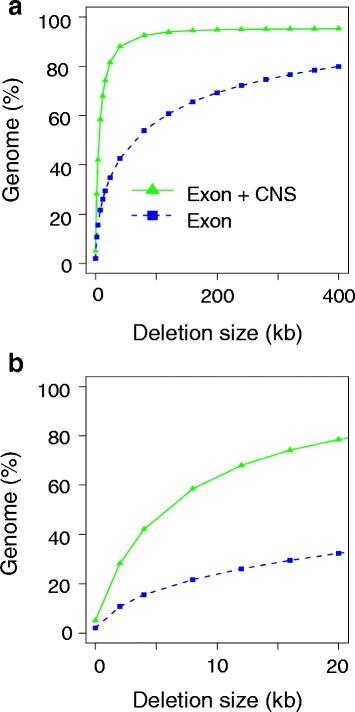
Proportion of genomic regions that can detect various deletion lengths. Two probe sets, TargetEC (green) and a subset of probes that correspond to exonic regions (blue), are used. **a** Deletion size up to 400 kb. **b** Zoom view to show detailed differences in deletion sizes up to 20 kb

By including CNSs in our target regions, the target density on the genome is much higher than the density achieved with conventional exome sequencing. In other words, the target capture kit can be used not only to detect SNVs and small INDELs within the target regions, but also to identify large deletions that contain target regions. To quantitatively assess the ability to detect large deletions using TargetEC, we measured the proportion of genomic regions in which large deletions of various lengths could be detected using the TargetEC probes, and compared the result with that of a hypothetically designed conventional exome probe that only targets exonic regions (Fig. 2). The probe intervals in TargetEC suggest that a 100-kb genomic deletion could be detected in 93.4 % of the entire genome, whereas this value decreased to 57.7 % using the conventional exome design. Similarly, a 10-kb genomic deletion could be detected in 63.8 % of the entire genome with TargetEC, whereas this value decreased to 24.1 % of the genome using the conventional exome design. We also measured the proportion of the CNSs that are located close vicinity to annotated exons. Those CNSs might be sequenced even by conventional exome design because of the insert size of the library in exome experiments (typically around 250 bp). We found that only 6.1 % of CNSs (41,393 out of 677,605 CNS regions) are located within 250 bp to the closest exons. This means that about 90 % of CNSs can not be covered by conventional exome design.

### Target capture sequencing

To evaluate the performance of TargetEC, we applied this probe set to four rat inbred strains (WTC/Kyo, WTC-*swh*/Kyo, PVG/Seac, and KFRS4/Kyo) maintained by the National BioResource Project for the Rat (NBRP-Rat) in Japan [32], followed by sequencing using an Illumina HiSeq 1500 with 2 × 101-bp or 2 × 251-bp paired-end sequencing (Table 1). The obtained reads have been deposited in a public database (DDBJ Sequence Read Archive accession number: DRA004543). WTC/Kyo and PVG/Seac were used as control strains for WTC-*swh*/Kyo and KFRS4/Kyo, respectively, both of the mutant strains exhibit prominent phenotypes (for details, see NBRP-Rat homepage: http://www.anim.med.kyoto-u.ac.jp/nbr/). The total numbers of bases obtained just after sequencing ranged from 33.2 Gb (PVG/Seac) to 37.6 Gb (WTC-*swh*/Kyo). These reads were mapped onto the rat genome (rn5; March 2012), and more than 99.1 % of the bases appeared to be mapped in all four samples. After removing duplicated reads, 85.2–94.2 % of the initially-obtained reads were retained as final mapped reads. Of these reads, more than 78.3 % of the initially-obtained reads were mapped onto target regions. Because we obtained a rather high number of reads, the average depth of coverage in the target regions ranged from 108.0 (WTC-*swh*/Kyo) to 125.3 (KFRS4/Kyo). In all four samples, more than 99 % of the target regions were covered with a depth of ≥10. We constructed plots to analyze the relationship between the depth of coverage and the target regions covered with a certain minimum depth (Additional file 1: Figure S1). For WTC/Kyo, 84.4 % of the target regions were covered by at least 50 reads, and 97.9 % by at least 20 reads. The coverage trends for the remaining three strains were very similar (Additional file 1: Figure S1). Here, we have to note that the median values of the insert size of the library range from 205 to 214 bp. This means that significant fraction of pairs of reads overlap with each other especially for the samples sequenced with the read length of 251 bp. We also measured the proportion of the target regions that was not covered by any reads in each of the four strains. This ranged from 0.15 to 0.17 %. The proportion of the target regions that are not covered by any reads in all four strains was only 0.067 %.

**Table 1:** Summary statistics for sequencing and mapping

Strain	Total reads	Read length	Mapped reads (%)	After removing duplicated reads (%)	Reads on target (%)	Average target depth	% target with coverage depth of ≥10×
WTC/Kyo	149,260,414	251	149,076,094 (99.9)	140,578,240 (94.2)	117,852,971 (79.0)	108.0	99.3
WTC-*swh*/Kyo	149,679,520	251	149,510,020 (99.9)	140,569,239 (93.9)	118,321,570 (79.0)	107.6	99.3
PVG/Seac	328,315,192	101	325,289,595 (99.1)	279,768,743 (85.2)	257,155,712 (78.3)	120.4	99.4
KFRS4/Kyo	335,799,700	101	333,078,486 (99.2)	289,224,244 (86.1)	264,672,454 (78.8)	125.3	99.4

According to the molecular phylogenetic trees of inbred laboratory rat strains constructed using microsatellite markers [33] and SNPs [23], WTC/Kyo, PVG/Seac, and the reference strain BN/SsNHsdMCW are widely separated on the tree, indicating a divergence in genomic sequences among these strains. Indeed, the polymorphic rate, measured by microsatellite markers, between WTC and BN/SsNHsdMCW was close to the highest value [33]. In spite of this divergence, the sequence reads of all strains analyzed in this study mapped well on the reference strain, BN/SsNHsdMCW. This indicates that although the probe sequences were designed based on the BN/SsNHsdMCW reference strain, TargetEC can be applied to a wide range of commonly-used inbred laboratory rat strains for the systematic identification of mutations in regions with functional importance (i.e., exons and CNSs).

### Variant identification

**Fig. 3:**
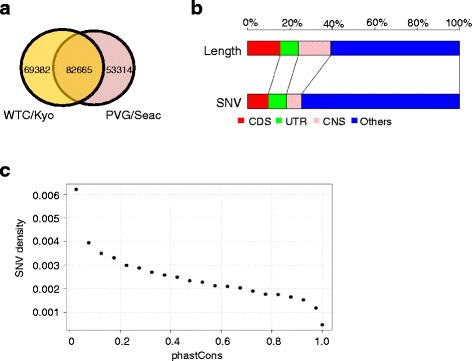
Variants identified by TargetEC. **a** Numbers of homozygous SNVs identified in the WTC/Kyo and PVG/Seac strains. Only SNVs in regions with a coverage depth of ≥10 were used. **b** Proportions of the number of SNVs in each class of target regions in the WTC/Kyo strain: CDS, UTR, CNS, and others. The proportions are compared with the region lengths. **c** The relationship between the phastCons conservation score and SNV density in the WTC/Kyo strain. SNV density was calculated as the number of SNVs divided by the lengths of regions with a certain phastCons score range (binned for every 0.05 interval)

We next applied variant calling procedures to mapped reads for SNV and INDEL identification (Table 2). Here we only considered mutations identified in target regions with a coverage depth of ≥10. In all groups of regions (i.e., CDS, UTR, and CNS), the numbers of SNVs and INDELs were higher in WTC/Kyo and WTC-swh/Kyo than in PVG/Seac and KFRS4/Kyo, which was derived as a congenic strain using PVG/Seac as an inbred partner strain. This finding indicates a higher level of genetic divergence in BN/SsNHsdMCW vs. WTC/Kyo than in BN/SsNHsdMCW vs. PVG/Seac. This result is consistent with the genomic differences measured using polymorphisms determined by 357 microsatellite markers, in which the rates of polymorphic markers in BN/SsNHsdMCW vs. WTC/Kyo and in BN/SsNHsdMCW vs. PVG/Seac were 89 and 85 %, respectively [33]. We also compared the SNVs identified in WTC/Kyo and PVG/Seac (Fig. 3a). We omitted the other two strains from this analysis as each was similar to either of the analyzed strains. We found that 54.4 % of SNVs in WTC/Kyo and 60.8 % of those in PVG/Seac were common between WTC/Kyo and PVG/Seac. The ratios of common SNVs in each group of regions (i.e., CDS, UTR, CNS, and other regions) were highly similar to those among the total identified SNVs (Additional file 1: Figure S2). The transition/transversion (Ti/Tv) ratio ranged from 3.56 to 3.66 in CDS, whereas the ranges for UTR and CNS were smaller (2.43–2.48 and 2.30–2.37, respectively). These values are consistent with previous reports [34]. The higher Ti/Tv ratio in CDS might be mainly attributed to codon degeneracy, i.e., the third position of a two-fold degenerate codon is always purine (A/G) or pyrimidine (C/T). We also calculated the numbers of mutations in various coverage depths (Additional file 1: Table S1).

**Table 2:** Summary statistics for SNV and INDEL

Strain	SNV				INDEL			
	Total^a^ (Ti/Tv)	CDS (Ti/Tv)	UTR (Ti/Tv)	CNS (Ti/Tv)	Total^a^	CDS	UTR	CNS
WTC/Kyo	152,047 (2.43)	14,943 (3.61)	12,932 (2.46)	10,816 (2.31)	28,915	775	2,744	3,101
WTC-*swh*/Kyo	151,981 (2.42)	14,942 (3.61)	12,953 (2.45)	10,824 (2.30)	28,870	753	2,727	3,124
PVG/Seac	135,979 (2.43)	13,554 (3.56)	11,778 (2.48)	9,494 (2.36)	25,876	719	2,538	2,737
KFRS4/Kyo	134,604 (2.43)	13,266 (3.66)	11,466 (2.43)	9,489 (2.37)	25,578	677	2,439	2,759

^a^Total numbers of SNV and INDEL include all of those in depth ≥10 regardless of being in target regions or not. Ti/Tv is the ratio of transitions to transversions

We also measured a proportion of the SNVs in terms of each target region class: CDS, UTR, CNS, and others (Fig. 3b and Additional file 1: Figure S3). Although we designed our capture kit to cover only exonic regions and CNSs (phastCons score = 1.0), it also covered some flanking regions because of the expansion to a minimum length of 100 bp and optimization of hybridization using the manufacturer’s internal protocols. Accordingly, approximately 60 % of the residues in the target could not be classified as CDS, UTR, or CNS. In Fig. 3b, we present our results for the WTC/Kyo strain, but note that the results for the other three strains were almost identical (Additional file 1: Figure S3). Our findings clearly indicate that mutations are highly suppressed among CNSs and CDSs, whereas fewer constraints are placed on UTR; in contrast, the remaining regions are relatively susceptible to mutations.

To further analyze the relationship between the mutation frequencies and selective pressure acting on each genomic region, we plotted SNV density as a function of the phastCons score (Fig. 3c and Additional file 1: Figure S4). As expected, we observed a clear negative correlation between the SNV density and phastCons score. Although phastCons scores are calculated for genome alignments of vertebrate species, which include not only mammals but also chicken and zebrafish, the existence of a negative correlation between the SNV density and phastCons score indicates a similar trend of selective pressure among different strains of rats.

### Validation of TargetEC for the identification of known causative mutations

To validate whether our approach could detect the causative mutations of certain phenotypes, we analyzed mutations identified by TargetEC in the WTC-*swh*/Kyo and KRFS4/Kyo strains. For each of these strains, we used WTC/Kyo and PVG/Seac, respectively, as control strains.

**Fig. 4:**
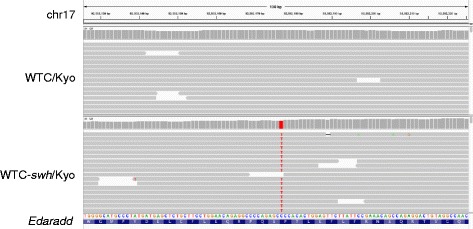
Validation of the causative mutation for HED in the WTC-*swh*/Kyo strain. A C-to-T transition mutation, which was previously confirmed as a causative mutation for HED [35], was identified in the *Edaradd* gene. The locus is compared with that of the normal strain (WTC/Kyo)

WTC-*swh*/Kyo is a rat model of hypohidrotic ectodermal dysplasia (HED) [35]. HED is known to be caused by mutations in genes encoding proteins involved in the Eda pathway, which include ectodysplasin (*Eda*), the ED receptor (*Edar*), and EDAR-associated death domain (*Edaradd*) [36]. In the case of WTC-*swh*/Kyo, previous linkage analysis revealed that the causative mutation resides in the distal end of rat chromosome 17 [27]. More recently, the locus was identified within a 161-kb interval between the microsatellite marker D17Rat140 (chr17:92,342,663) and the telomere [35]. We identified a total of 151,981 SNVs and 28,870 INDELs. After subtracting the known variants registered in dbSNP (Build 138; April 25, 2013), 46,737 SNVs and 28,378 INDELs remained. These variants were further filtered using information from the linkage analysis, yielding a final total of two SNVs and two INDELs. One of these was a non-synonymous variant in the CDS of *Edaradd*. By evaluating the functional protein-level consequences of these mutations using the PolyPhen-2 program [37], the C to T transition mutation in the last exon of *Edaradd* (Fig. 4), which changes proline to serine at codon 153 of the EDARADD protein, was predicted to be a damaging mutation. Indeed, this mutation was previously identified as causative for the HED phenotype [35].

**Fig. 5:**
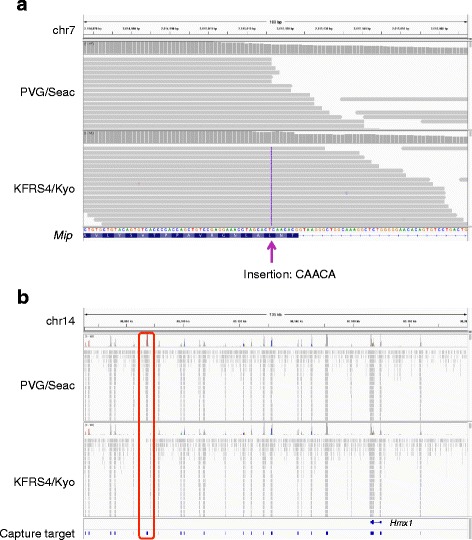
Validation of mutations in the KFRS4/Kyo strain. Each locus is compared with the corresponding locus in the closely related strain PVG/Seac. **a** The causative mutation for cataracts. A 5-bp insertion, which was previously reported as the causative mutation for cataracts in KFRS4/Kyo [28], was identified near the end of exon 1 of the *Mip* gene. **b** Deletion of a CNS responsible for congenital ear malformation in KFRS4/Kyo. This deletion, which was previously identified as the mutation responsible for congenital ear malformation [29], was located approximately 80-kb downstream of the *Hmx1* gene

Next, we sought to validate whether this method could successfully detect the causative mutation for cataract development in the KFRS4/Kyo strain [28]. By linkage analysis, the mutation responsible for the cataract phenotype was mapped to an interval of 9.7 Mb on chromosome 7 [28], which contains about 150 genes. We identified 134,604 SNVs and 25,578 INDELs in the KFRS4/Kyo strain. The numbers of variants were reduced to 49,747 SNVs and 25,190 INDELs after filtering out known variants. Among these, 234 SNVs and 72 INDELs were located in the 9.7-Mb interval identified by the linkage analysis. *Mip*, one of the genes in this region, has previously been identified as a causative gene because of its known role in cataracts in humans and mice [38, 39], and an observed 5-bp insertion in KFRS4/Kyo was predicted to have a severe effect on the protein product [28]. Among the 72 INDELs identified by the TargetEC reads obtained for KFRS4/Kyo, we also observed the same 5-bp insertion in exon 1 of *Mip* (Fig. 5a), indicating that the TargetEC method can detect the causative mutation for cataract formation in KFRS4/Kyo.

The KFRS4/Kyo strain also exhibits other phenotypes [40]. One such phenotype is congenital ear malformation, also known as the “dumbo” phenotype [40]. Although the responsible locus was mapped to a 6-Mb interval on chromosome 14, which contains the *Hmx1* gene, no coding changes were identified in this region [40]; eventually, deletion of a conserved regulatory element for *Hmx1*, located approximately 80-kb downstream of the gene, was identified [29]. Here, we applied the MACS2 program, which is usually used for peak calling in ChIP-seq experiments, to the mapped reads of KFRS4/Kyo and the control strain PVG/Seac in order to systematically identify large deletions as differences in the accumulated reads. We identified 6554 peak regions specific for PVG/Seac, which were hence thought to be KFRS4/Kyo-specific deletions. Of these peaks, 15 were located within the 6.0-Mb interval on chromosome 14; the most significant, according to the ratio of read numbers, was the deletion located approximately 80-kb downstream of *Hmx1* (Fig. 5b), which exactly corresponds to the deletion identified in the conserved regulatory element for *Hmx1* [29].

## Discussion

In this paper, we designed a novel target capture probe set, TargetEC, for rat genome analysis. This probe set not only covers exons for all annotated genes but also covers regions that are highly conserved across vertebrate species. Although the span of covered regions (146.8 Mb) is approximately three times larger than that covered by conventional human (58.0 Mb; Agilent Human All Exon V6) and mouse (49.6 Mb; Agilent Mouse All Exon) exome probes, it can potentially identify regulatory mutations that cannot be detected using conventional exome strategies. Another advantage associated with the inclusion of conserved regions is that most genic regions will be covered even without explicitly using gene annotation information. This can be a great advantage, especially for organisms with poorly annotated genes.

Regarding variants identified in coding regions, predictions of functional consequences are rather straightforward because several types of mutations, including synonymous, non-synonymous, inframe stop, and frameshift mutations exist, and each has different effects on the protein products. Indeed, several existing programs can classify these types of mutations [41] and predict the severity of the mutant phenotypes [37, 42, 43]. On the other hand, predicting the effects of variations in non-coding regions might not be an easy task. This might explain why non-coding regions have not been previously analyzed using conventional target capture methods (i.e., exome analysis), although a certain fraction of disease-associated mutations are known to be located in non-coding regions (for review, see [15–18]). In this study, we included both CNSs and exons as capture targets. These CNSs may be plausible targets for regulatory mutations because of the high conservation among vertebrate species, which implies the action of strong negative selection on these sites. Validation of the ability of our target capture kit to detect mutations in the regulatory element of *Hmx1* suggests that this kit can be a powerful tool for the detection of regulatory mutations, which are not currently well annotated in genome databases. To evaluate the functional relevance of CNS mutations in the rat, we can incorporate information about epigenomic states [44, 45] such as H3K4me1, the histone modification associated with enhancers in the human and mouse, by transferring this information to corresponding regions in the rat genome [46]. Given the number and the total length of CNSs, huge amounts of regulatory mutations might be responsible for certain phenotypes because mutations in non-coding regions only alter the spatio-temporal expression of target genes while maintaining the function of the protein products. On the other hand, critical mutations in coding regions completely change the functions of protein products, which might lead to severe or often lethal phenotypes.

In conventional exome analysis, information about SNPs helps to narrow down the number of candidate mutations. In humans, the number of SNPs registered in dbSNP has increased rapidly, and continues to increase further as a result of progress in genome sequencing projects for individuals such as the 1000 Genomes Project [47]. On the other hand, the number of SNPs identified in rat is far less than those in the human and mouse. However, recent continuous and steady progress has been made in the field of rat genome resequencing [23–26]. The genome resequencing of closely-related reference strains has yielded massive and valuable information about genetic variations in the rat. Exome sequencing of many sample strains represents another possible approach to overcoming this shortfall of information about genetic variations in the rat. In fact, this strategy was adopted in the pilot phase of the 1000 Genomes Project, where more than half of the samples were subjected to exome sequencing to reveal rare variations in a cost-effective manner [48]. Following this strategy, the application of TargetEC to a large stock of mutant strains, such as that stored by NBRP-Rat in Japan [32], would be useful for the collection of common and rare variations in functionally important regions and would therefore facilitate the identification of mutations responsible for certain phenotypes in mutant strains. This type of analysis is our future aim. The data obtained during this analytical course would be disclosed to public repositories, and would thus be of potential value to the rat genetics and genomics research communities.

One of the drawbacks for TargetEC is that the total length of the covered regions is approximately three-fold greater than that achieved with a conventional exome kit (e.g., a widely used human exome capture kit). Although a three-fold higher number of reads is needed to obtain the same depth of coverage as those obtained by conventional exome sequencing, the total length of the target regions remains 20-fold less than the entire genome, thus confirming the feasibility of this strategy relative to whole genome sequencing (WGS) when seeking relevant mutations for certain phenotypes. Moreover, rapid growth in sequencer performance will render the TargetEC strategy an even more affordable option for the detection of variations not only in exonic regions, but also in functionally important CNSs.

To experimentally confirm the involvement of a candidate mutation identified by target capture sequencing, we can use a gene knockout procedure that was recently established for rats. An early *N*-ethyl-*N*-nitrosourea (ENU) mutagenesis-based method was devised for knockout rat generation [49, 50]. With the recent emergence of genome editing technology, it is more feasible to implement genomic modifications in rats to analyze the phenotypic effects of genes [51, 52]. In these circumstances, TargetEC can be applied to laboratory rats that exhibit various disease phenotypes; the resulting experimental confirmation of the involvement of the identified mutations would shed light on the genetic cause underlying these mutants and promote mechanistic insights into the disease etiology.

This design strategy (i.e., targeting both exonic regions and CNSs) can easily be applied to human samples for the detection of regulatory mutations associated with certain phenotypes. Moreover, TargetEC developed in this study might have a potential to be directly applied to cross-species capture [11], for example, for other rodents, because it covers all the regions that are highly conserved (phastCons score = 1.0) among vertebrates, although we need further evaluation of experimental conditions and performance for such purpose [53].

## Conclusions

The target capture design reported herein can potentially be used to identify additional mutations responsible for various phenotypes in the rat, which is a suitable model organism for comparative studies of diseases and other traits. The potential mutations include not only those in coding regions but also those in CNSs, which might correspond to regulatory mutations. By further analyzing these mutations, we will deepen our understanding of the relationships between the genotypic and phenotypic variations associated with regulatory elements.

## Methods

### Animals

WTC/Kyo (NBRP Rat No. 0020), WTC-*swh*/Kyo (NBRP Rat No. 0287), PVG/Seac (NBRP Rat No. 0080), and KFRS4/Kyo (NBRP Rat No. 0572) rats were provided by the Japanese NBRP-Rat [32]; http://www.anim.med.kyoto-u.ac.jp/nbr/) and maintained at the Institute of Laboratory Animals, Graduate School of Medicine, Kyoto University, under a specific pathogen-free environment. All animal experimentation protocols were approved by the Institutional Animal Care and Use Committees of Kyoto University and were conducted according to the Regulation on Animal Experimentation at Kyoto University.

### Genome annotation data used to design the target regions for TargetEC

The rat genome assembly rn5 (RGSC 5.0, March 2012) was used as a reference genome in this study. We used gene annotations from RefSeq [30] and Ensembl [31] for the rat (rn5); these were downloaded from the Table Browser function in the UCSC Genome Browser [54]. We also used highly conserved regions (phastCons score = 1.0) [21] among 13 vertebrate species (multiz alignment of 13 vertebrates), which were also downloaded from the UCSC Genome Browser [55]. We merged all of these regions (i.e., those annotated in RefSeq and/or Ensembl and the conserved regions) using the “merge” option in the BEDTools program [56]. Regions <100 bp in length were expanded in both directions to reach a minimum length of 100 bp. After these expansions, overlapping regions were further unified using the “merge” option in the BEDTools program. Based on the obtained target regions, a probe set for sequence capture (SeqCap EZ Developer Library; Roche NimbleGen, Madison, WI, USA) was designed and synthesized according to the manufacturer’s internal protocols.

### Sample preparation and sequencing

Target capture was performed using the standard SeqCap EZ System protocol (Roche NimbleGen). DNA sequencing libraries were prepared using the KAPA HyperPlus Library Preparation Kit (KAPA Biosystems, London, UK) according to the manufacturer’s protocol. We applied two samples for one-reaction reagents. Sequencing was performed on an Illumina HiSeq 1500 (Illumina, San Diego, CA, USA) in Rapid Run mode PE250 (v2) (WTC/Kyo and WTC-*swh*/Kyo) and Rapid Run mode PE100 (v1) (PVG/Seac and KFRS4/Kyo), according to the manufacturer’s protocol.

### Quality control of the sequencing reads

The reads generated using the Illumina HiSeq platform were subjected to quality control to exclude low quality reads. Initially, we used the FastQC program (http://www.bioinformatics.babraham.ac.uk/projects/fastqc/) to evaluate the overall read quality. Next, we trimmed reads from the 3’-end to address low quality bases (quality score of <30). After this trimming step, shortened reads (length of <30) were discarded from further analysis.

### Read mapping and variant identification

We followed a previously reported basic exome data analysis procedure [57]. In brief, the reads that passed the above quality control were first mapped onto the reference rat genome (rn5; RGSC 5.0, March 2012) using BWA (v0.7.4) [58] with the default parameters. SAMtools (v0.1.12a) [59] was used for file format conversion and indexing. For post-processing of this mapping step, duplicated reads were removed based on mapping position of the mates using the MarkDuplicates program in Picard tools (v1.87) (http://broadinstitute.github.io/picard/). Next, the RealignerTargetCreator and IndelRealigner utilities in the Genome Analysis Toolkit (GATK; v3.6) [60] and the FixMateInformation utility in Picard tools were used to realign the reads. To calculate basic statistics, we considered a read to be on target if the read overlap a target region by at least 1 bp. Quality score recalibration was conducted with the BaseRecalibrator utility in the GATK, using known SNVs and small INDELs collected from dbSNP (Build 138; April 25, 2013). The same known variant set was used for filtering to narrow down the number of candidate mutations associated with the phenotypes. Variant calling was performed using the HaplotypeCaller utility in the GATK.

### Variant annotation

BEDTools (v2.17.0) [56] was used to filter out known variants from those identified in a sample. ANNOVAR (version 22-March-2015) [41] was used for the functional annotation of variants, with RefSeq genes and Ensembl genes used as the rat gene annotations. PolyPhen-2 was used to assess the functional consequences of mutations [37]. The Integrative Genome Viewer (IGV; v2_2_13) [61] was used to visualize the mapped reads and variants.

### Detection of large deletions

The BAM files obtained from the read mapping were used to detect large deletions. For this procedure, we applied the peak calling program, MACS2 [62], to the BAM file for the mutant strain and its control strain. By comparing the peak regions in the mutant and control strains using the Subtract option in BEDTools [56], we defined a deletion in the mutant strain if there is a peak only in the control strain. Then the identified peaks were visually inspected by using IGV (v2_2_13) [61].

## Abbreviations

CNS, conserved non-coding sequence; GAWS, genome-wide association study; SNV, single nucleotide variant; WES, whole exome sequencing.

## Additional file

Additional file 1:
**Table S1.** Summary statistics for SNV and INDEL in various depths. **Figure S1.** Sequence coverage of the target regions for each rat strain. **Figure S2.** Number of homozygous SNVs identified in WTC/Kyo and PVG/Seac strains for each genomic region. **Figure S3.** Proportion of the number of SNVs in terms of the each class of regions in the target, i.e., CDS, UTR, CNS, and other regions, for each rat strain. **Figure S4.** The relationship between the phastCons conservation score and SNV density for each rat strain. (PDF 215 kb)

## References

[1] Hodges E, Xuan Z, Balija V, Kramer M, Molla MN, Smith SW (2007). Genome-wide in situ exon capture for selective resequencing. Nat Genet.

[2] Ng SB, Buckingham KJ, Lee C, Bigham AW, Tabor HK, Dent KM (2010). Exome sequencing identifies the cause of a mendelian disorder. Nat Genet.

[3] Rabbani B, Tekin M, Mahdieh N (2014). The promise of whole-exome sequencing in medical genetics. J Hum Genet.

[4] Fairfield H, Gilbert GJ, Barter M, Corrigan RR, Curtain M, Ding Y (2011). Mutation discovery in mice by whole exome sequencing. Genome Biol.

[5] Fairfield H, Srivastava A, Ananda G, Liu R, Kircher M, Lakshminarayana A (2015). Exome sequencing reveals pathogenic mutations in 91 strains of mice with Mendelian disorders. Genome Res.

[6] Gibbs RA, Weinstock GM, Metzker ML, Muzny DM, Sodergren EJ, Scherer S (2004). Genome sequence of the Brown Norway rat yields insights into mammalian evolution. Nature.

[7] Broeckx BJG, Coopman F, Verhoeven GEC, Bavegems V, De Keulenaer S, De Meester E (2014). Development and performance of a targeted whole exome sequencing enrichment kit for the dog (Canis Familiaris Build 3.1). Sci Rep.

[8] Broeckx BJG, Hitte C, Coopman F, Verhoeven GEC, De Keulenaer S, De Meester E (2015). Improved canine exome designs, featuring ncRNAs and increased coverage of protein coding genes. Sci Rep.

[9] Robert C, Fuentes-Utrilla P, Troup K, Loecherbach J, Turner F, Talbot R (2014). Design and development of exome capture sequencing for the domestic pig (Sus scrofa). BMC Genomics.

[10] Cosart T, Beja-Pereira A, Chen S, Ng SB, Shendure J, Luikart G (2011). Exome-wide DNA capture and next generation sequencing in domestic and wild species. BMC Genomics.

[11] Bi K, Vanderpool D, Singhal S, Linderoth T, Moritz C, Good JM (2012). Transcriptome-based exon capture enables highly cost-effective comparative genomic data collection at moderate evolutionary scales. BMC Genomics.

[12] Jin X, He M, Ferguson B, Meng Y, Ouyang L, Ren J (2012). An effort to use human-based exome capture methods to analyze chimpanzee and macaque exomes. PLoS One.

[13] Lettice LA, Heaney SJH, Purdie LA, Li L, de Beer P, Oostra BA (2003). A long-range Shh enhancer regulates expression in the developing limb and fin and is associated with preaxial polydactyly. Hum Mol Genet.

[14] Benko S, Fantes JA, Amiel J, Kleinjan D-J, Thomas S, Ramsay J (2009). Highly conserved non-coding elements on either side of SOX9 associated with Pierre Robin sequence. Nat Genet.

[15] Epstein DJ (2009). Cis-regulatory mutations in human disease. Brief Funct Genomic Proteomic.

[16] Ward LD, Kellis M (2012). Interpreting noncoding genetic variation in complex traits and human disease. Nat Biotechnol.

[17] Douglas AT, Hill RD (2014). Variation in vertebrate cis-regulatory elements in evolution and disease. Transcription.

[18] Mathelier A, Shi W, Wasserman WW (2015). Identification of altered cis-regulatory elements in human disease. Trends Genet TIG.

[19] Welter D, Macarthur J, Morales J, Burdett T, Hall P, Junkins H (2014). The NHGRI GWAS Catalog, a curated resource of SNP-trait associations. Nucleic Acids Res.

[20] Sakabe NJ, Savic D, Nobrega MA (2012). Transcriptional enhancers in development and disease. Genome Biol.

[21] Siepel A, Bejerano G, Pedersen JS, Hinrichs AS, Hou M, Rosenbloom K (2005). Evolutionarily conserved elements in vertebrate, insect, worm, and yeast genomes. Genome Res.

[22] Sherry ST, Ward MH, Kholodov M, Baker J, Phan L, Smigielski EM (2001). dbSNP: the NCBI database of genetic variation. Nucleic Acids Res.

[23] Saar K, Beck A, Bihoreau M-T, Birney E, Brocklebank D, STAR Consortium (2008). SNP and haplotype mapping for genetic analysis in the rat. Nat Genet.

[24] Atanur SS, Diaz AG, Maratou K, Sarkis A, Rotival M, Game L (2013). Genome sequencing reveals loci under artificial selection that underlie disease phenotypes in the laboratory rat. Cell.

[25] Baud A, Hermsen R, Guryev V, Stridh P, Graham D, Rat Genome Sequencing and Mapping Consortium (2013). Combined sequence-based and genetic mapping analysis of complex traits in outbred rats. Nat Genet.

[26] Hermsen R, de Ligt J, Spee W, Blokzijl F, Schäfer S, Adami E (2015). Genomic landscape of rat strain and substrain variation. BMC Genomics.

[27] Kuramoto T, Morimura K, Nomoto T, Namiki C, Hamada S, Fukushima S (2005). Sparse and wavy hair: a new model for hypoplasia of hair follicle and mammary glands on rat chromosome 17. J Hered.

[28] Watanabe K, Wada K, Ohashi T, Okubo S, Takekuma K, Hashizume R (2012). A 5-bp insertion in Mip causes recessive congenital cataract in KFRS4/Kyo rats. PLoS One.

[29] Quina LA, Kuramoto T, Luquetti DV, Cox TC, Serikawa T, Turner EE (2012). Deletion of a conserved regulatory element required for Hmx1 expression in craniofacial mesenchyme in the dumbo rat: a newly identified cause of congenital ear malformation. Dis Model Mech.

[30] Pruitt KD, Brown GR, Hiatt SM, Thibaud-Nissen F, Astashyn A, Ermolaeva O (2014). RefSeq: an update on mammalian reference sequences. Nucleic Acids Res.

[31] Cunningham F, Amode MR, Barrell D, Beal K, Billis K, Brent S (2015). Ensembl 2015. Nucleic Acids Res.

[32] Serikawa T, Mashimo T, Takizawa A, Okajima R, Maedomari N, Kumafuji K (2009). National BioResource Project-Rat and related activities. Exp Anim Jpn Assoc Lab Anim Sci.

[33] Mashimo T, Voigt B, Tsurumi T, Naoi K, Nakanishi S, Yamasaki K (2006). A set of highly informative rat simple sequence length polymorphism (SSLP) markers and genetically defined rat strains. BMC Genet.

[34] Schaibley VM, Zawistowski M, Wegmann D, Ehm MG, Nelson MR, St Jean PL (2013). The influence of genomic context on mutation patterns in the human genome inferred from rare variants. Genome Res.

[35] Kuramoto T, Yokoe M, Hashimoto R, Hiai H, Serikawa T (2011). A rat model of hypohidrotic ectodermal dysplasia carries a missense mutation in the Edaradd gene. BMC Genet.

[36] Sadier A, Viriot L, Pantalacci S, Laudet V (2014). The ectodysplasin pathway: from diseases to adaptations. Trends Genet TIG.

[37] Adzhubei IA, Schmidt S, Peshkin L, Ramensky VE, Gerasimova A, Bork P (2010). A method and server for predicting damaging missense mutations. Nat Methods.

[38] Shiels A, Bassnett S (1996). Mutations in the founder of the MIP gene family underlie cataract development in the mouse. Nat Genet.

[39] Varadaraj K, Kumari SS, Patil R, Wax MB, Mathias RT (2008). Functional characterization of a human aquaporin 0 mutation that leads to a congenital dominant lens cataract. Exp Eye Res.

[40] Kuramoto T, Yokoe M, Yagasaki K, Kawaguchi T, Kumafuji K, Serikawa T (2010). Genetic analyses of fancy rat-derived mutations. Exp Anim Jpn Assoc Lab Anim Sci.

[41] Wang K, Li M, Hakonarson H (2010). ANNOVAR: functional annotation of genetic variants from high-throughput sequencing data. Nucleic Acids Res.

[42] Kumar P, Henikoff S, Ng PC (2009). Predicting the effects of coding non-synonymous variants on protein function using the SIFT algorithm. Nat Protoc.

[43] Buchan DWA, Minneci F, Nugent TCO, Bryson K, Jones DT (2013). Scalable web services for the PSIPRED Protein Analysis Workbench. Nucleic Acids Res.

[44] ENCODE Project Consortium (2012). An integrated encyclopedia of DNA elements in the human genome. Nature.

[45] Stamatoyannopoulos JA, Snyder M, Hardison R, Ren B, Gingeras T, Mouse ENCODE Consortium (2012). An encyclopedia of mouse DNA elements (Mouse ENCODE). Genome Biol.

[46] Hinrichs AS, Karolchik D, Baertsch R, Barber GP, Bejerano G, Clawson H (2006). The UCSC Genome Browser Database: update 2006. Nucleic Acids Res.

[47] Auton A, Brooks LD, Durbin RM, Garrison EP, Kang HM, 1000 Genomes Project Consortium (2015). A global reference for human genetic variation. Nat.

[48] Abecasis GR, Altshuler D, Auton A, Brooks LD, Durbin RM, 1000 Genomes Project Consortium (2010). A map of human genome variation from population-scale sequencing. Nat.

[49] Zan Y, Haag JD, Chen K-S, Shepel LA, Wigington D, Wang Y-R (2003). Production of knockout rats using ENU mutagenesis and a yeast-based screening assay. Nat Biotechnol.

[50] Smits BMG, Mudde JB, van de Belt J, Verheul M, Olivier J, Homberg J (2006). Generation of gene knockouts and mutant models in the laboratory rat by ENU-driven target-selected mutagenesis. Pharmacogenet Genomics.

[51] Geurts AM, Cost GJ, Freyvert Y, Zeitler B, Miller JC, Choi VM (2009). Knockout rats via embryo microinjection of zinc-finger nucleases. Science.

[52] Mashimo T, Takizawa A, Voigt B, Yoshimi K, Hiai H, Kuramoto T (2010). Generation of knockout rats with X-linked severe combined immunodeficiency (X-SCID) using zinc-finger nucleases. PLoS One.

[53] Paijmans JLA, Fickel J, Courtiol A, Hofreiter M, Förster DW (2016). Impact of enrichment conditions on cross-species capture of fresh and degraded DNA. Mol Ecol Resour.

[54] Karolchik D, Hinrichs AS, Furey TS, Roskin KM, Sugnet CW, Haussler D (2004). The UCSC Table Browser data retrieval tool. Nucleic Acids Res.

[55] Rosenbloom KR, Armstrong J, Barber GP, Casper J, Clawson H, Diekhans M (2015). The UCSC Genome Browser database: 2015 update. Nucleic Acids Res.

[56] Quinlan AR, Hall IM (2010). BEDTools: a flexible suite of utilities for comparing genomic features. Bioinformatics.

[57] Altmann A, Weber P, Bader D, Preuss M, Binder EB, Müller-Myhsok B (2012). A beginners guide to SNP calling from high-throughput DNA-sequencing data. Hum Genet.

[58] Li H, Durbin R (2009). Fast and accurate short read alignment with Burrows-Wheeler transform. Bioinformatics.

[59] Li H, Handsaker B, Wysoker A, Fennell T, Ruan J, Homer N (2009). The Sequence Alignment/Map format and SAMtools. Bioinformatics.

[60] McKenna A, Hanna M, Banks E, Sivachenko A, Cibulskis K, Kernytsky A (2010). The Genome Analysis Toolkit: a MapReduce framework for analyzing next-generation DNA sequencing data. Genome Res.

[61] Thorvaldsdóttir H, Robinson JT, Mesirov JP (2013). Integrative Genomics Viewer (IGV): high-performance genomics data visualization and exploration. Brief Bioinform.

[62] Feng J, Liu T, Qin B, Zhang Y, Liu XS (2012). Identifying ChIP-seq enrichment using MACS. Nat Protoc.

