# Supplementary material for: Design and application of a target capture sequencing of exons and conserved non-coding sequences for the rat
Source: BMC Genomics. 2016 Aug 9;17:593. doi: 10.1186/s12864-016-2975-9 (PMC4979189; doi:10.1186/s12864-016-2975-9)
Supplement: Additional file 1: — Table S1. Summary statistics for SNV and INDEL in various depths. Figure S1. Sequence coverage of the target regions for each rat strain. Figure S2. Number of homozygous SNVs identified in WTC/Kyo and PVG/Seac strains for each genomic region. Figure S3. Proportion of the number of SNVs in terms of the each class of regions in the target, i.e., CDS, UTR, CNS, and other regions, for each rat strain. Figure S4. The relationship between the phastCons conservation score and SNV density for each rat strain. (PDF 215 kb) [file 12864_2016_2975_MOESM1_ESM.pdf]

Table S1. Summary statistics for SNV and INDEL in various depths

Depth	Strain	SNV				INDEL			
		Total[a] (Ti/Tv)	CDS (Ti/Tv)	UTR (Ti/Tv)	CNS (Ti/Tv)	Total[a]	CDS	UTR	CNS
≥25	WTC/Kyo	145,602 (2.47)	14,192 (3.74)	12,466 (2.51)	10,618 (2.33)	26,872	695	2,612	2,974
	WTC-*swh*/Kyo	145,458 (2.47)	14,164 (3.73)	12,439 (2.51)	10,615 (2.33)	26,874	670	2,580	3,008
	PVG/Seac	131,760 (2.47)	13,007 (3.66)	11,405 (2.54)	9,384 (2.37)	24,576	643	2,438	2,669
	KFRS4/Kyo	130,901 (2.47)	12,831 (3.75)	11,118 (2.47)	9,388 (2.38)	24,409	623	2,330	2,698
≥50	WTC/Kyo	122,132 (2.49)	11,300 (3.81)	11,339 (2.59)	10,005 (2.33)	21,892	513	2,319	2,706
	WTC-*swh*/Kyo	121,933 (2.48)	11,235 (3.87)	11,290 (2.60)	10,012 (2.33)	21,837	490	2,295	2,703
	PVG/Seac	116,430 (2.50)	11,194 (3.78)	10,697 (2.61)	9,076 (2.39)	21,054	497	2,202	2,501
	KFRS4/Kyo	117,209 (2.49)	11,182 (3.81)	10,480 (2.56)	9,114 (2.39)	21,150	507	2,148	2,534
≥75	WTC/Kyo	89,657 (2.47)	7,584 (3.75)	9,520 (2.60)	8,771 (2.32)	16,079	351	1,914	2,273
	WTC-*swh*/Kyo	89,172 (2.46)	7,424 (3.80)	9,487 (2.63)	8,809 (2.31)	16,079	346	1,908	2,289
	PVG/Seac	90,635 (2.50)	8,276 (3.78)	9,470 (2.63)	8,257 (2.39)	16,199	387	1,905	2,181
	KFRS4/Kyo	93,223 (2.50)	8,538 (3.85)	9,386 (2.58)	8,377 (2.40)	16,720	393	1,872	2,261

Ti/Tv is the ratio of transitions to transversions.

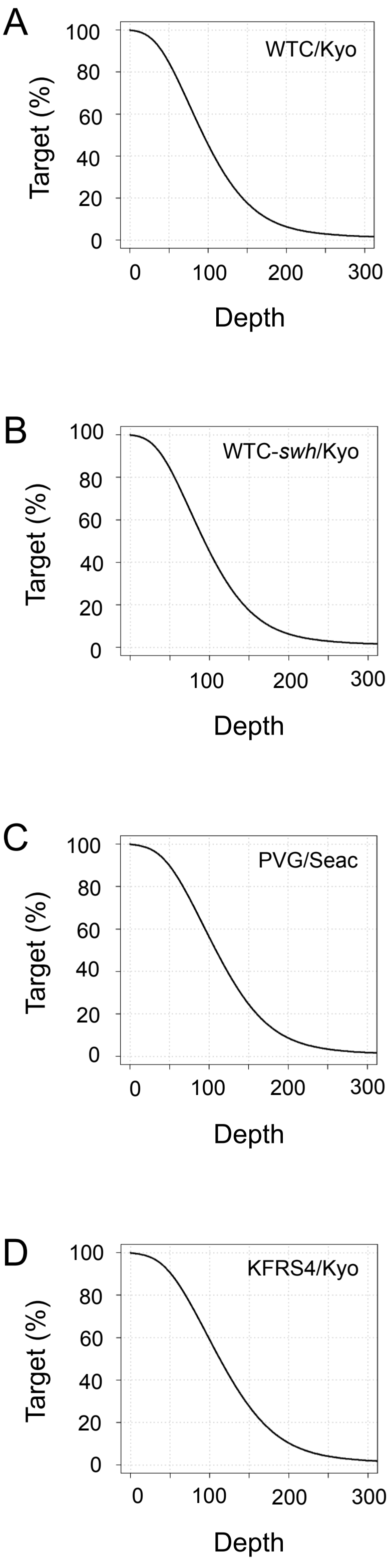

Figure S1. Sequence coverage of the target regions for each rat strain. Minimum depth of coverage is shown on the X-axis, and percentage of the residues in the target regions is shown on the Y-axis. (A) WTC/Kyo, (B) WTC-*swh*/Kyo, (C) PVG/Seac, and (D) KFRS4/Kyo.

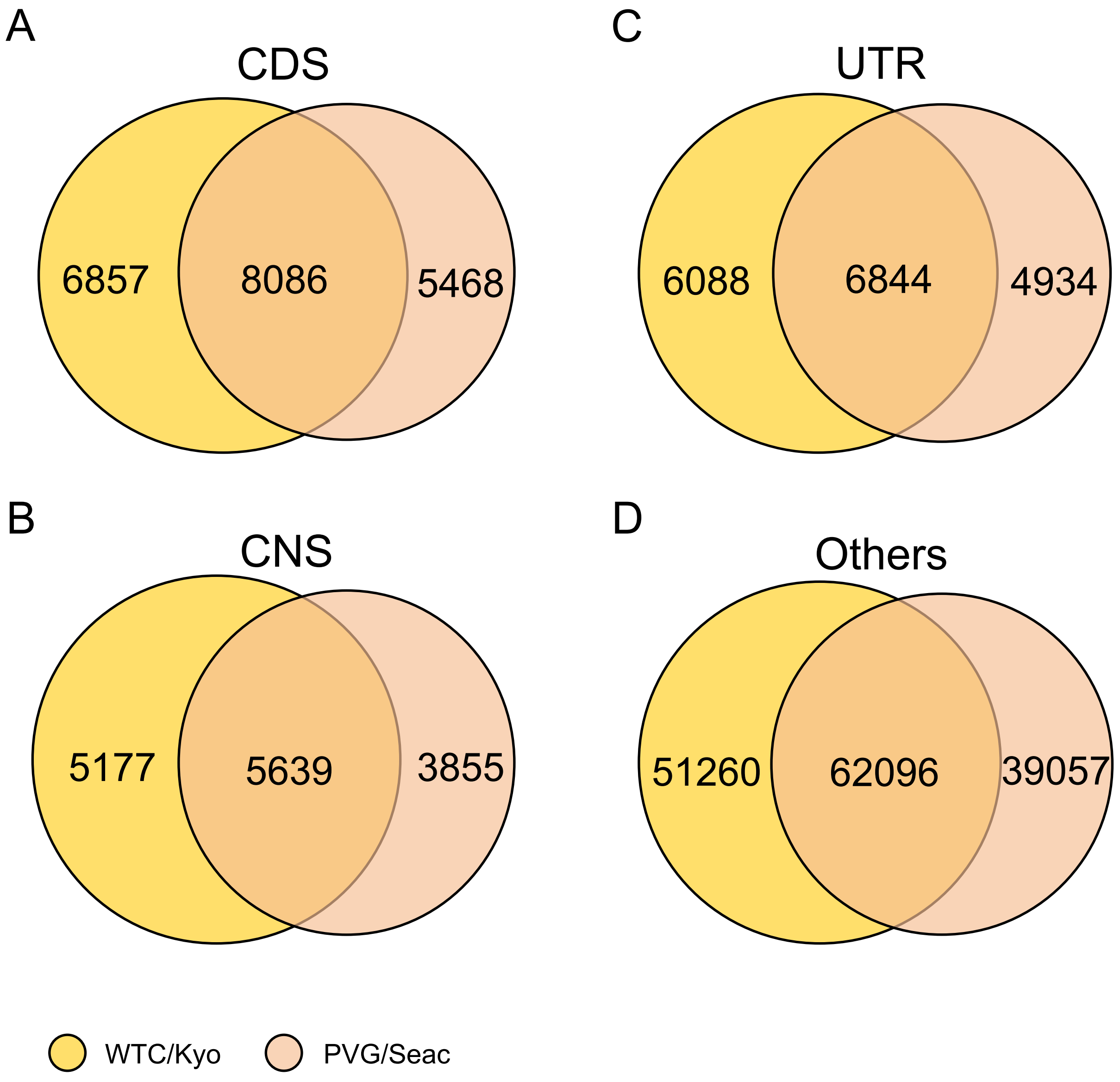

Figure S2. Number of homozygous SNVs identified in WTC/Kyo and PVG/Seac strains for each genomic region. (A) CDS, (B) CNS, (C) UTR, and (D) other regions.

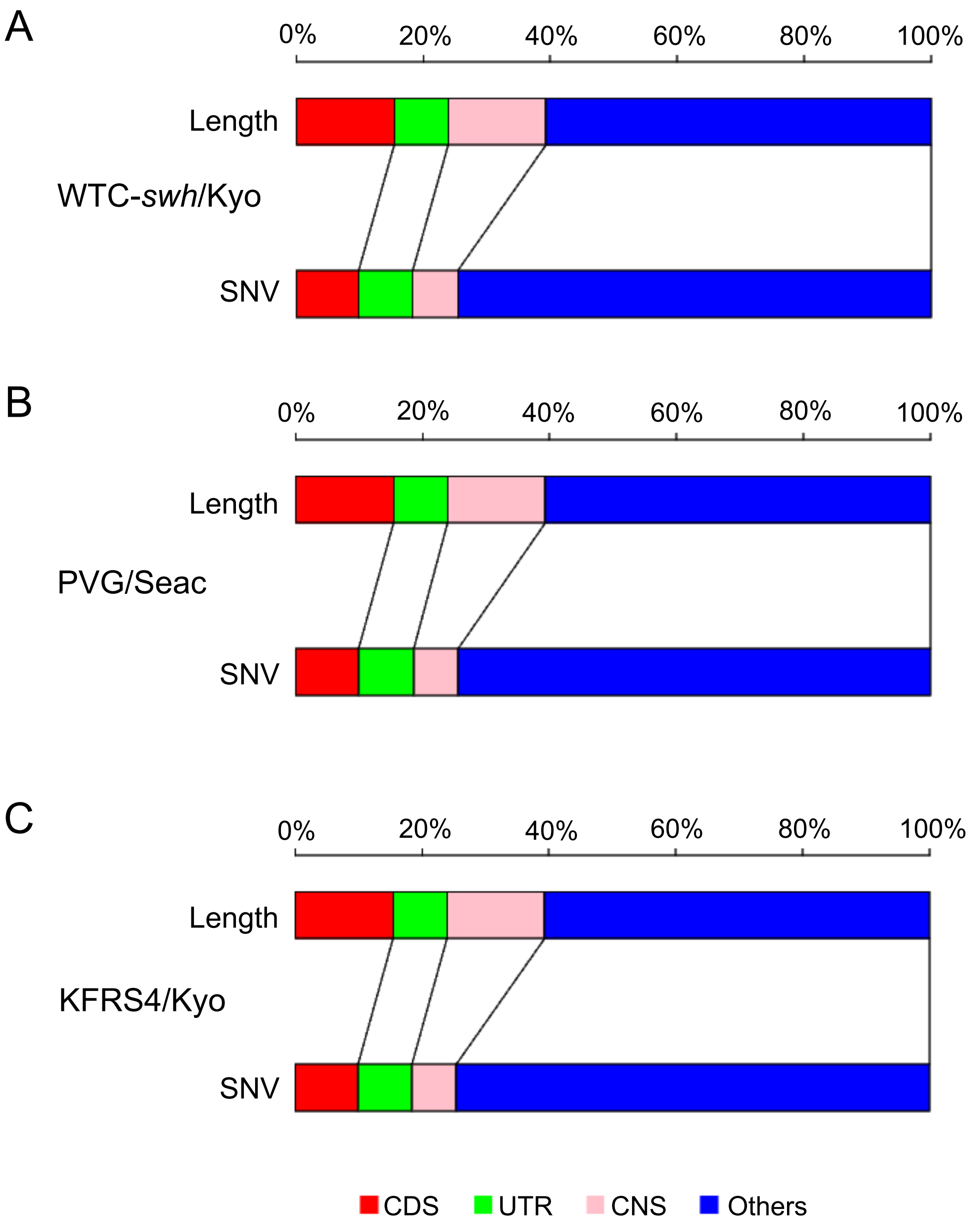

Figure S3. Proportion of the number of SNVs in terms of the each class of regions in the target, i.e., CDS, UTR, CNS, and other regions, for each rat strain. The proportion is compared with the length of the regions. (A) WTC-*swh*/Kyo, (B) PVG/Seac, and (C) KFRS4/Kyo.

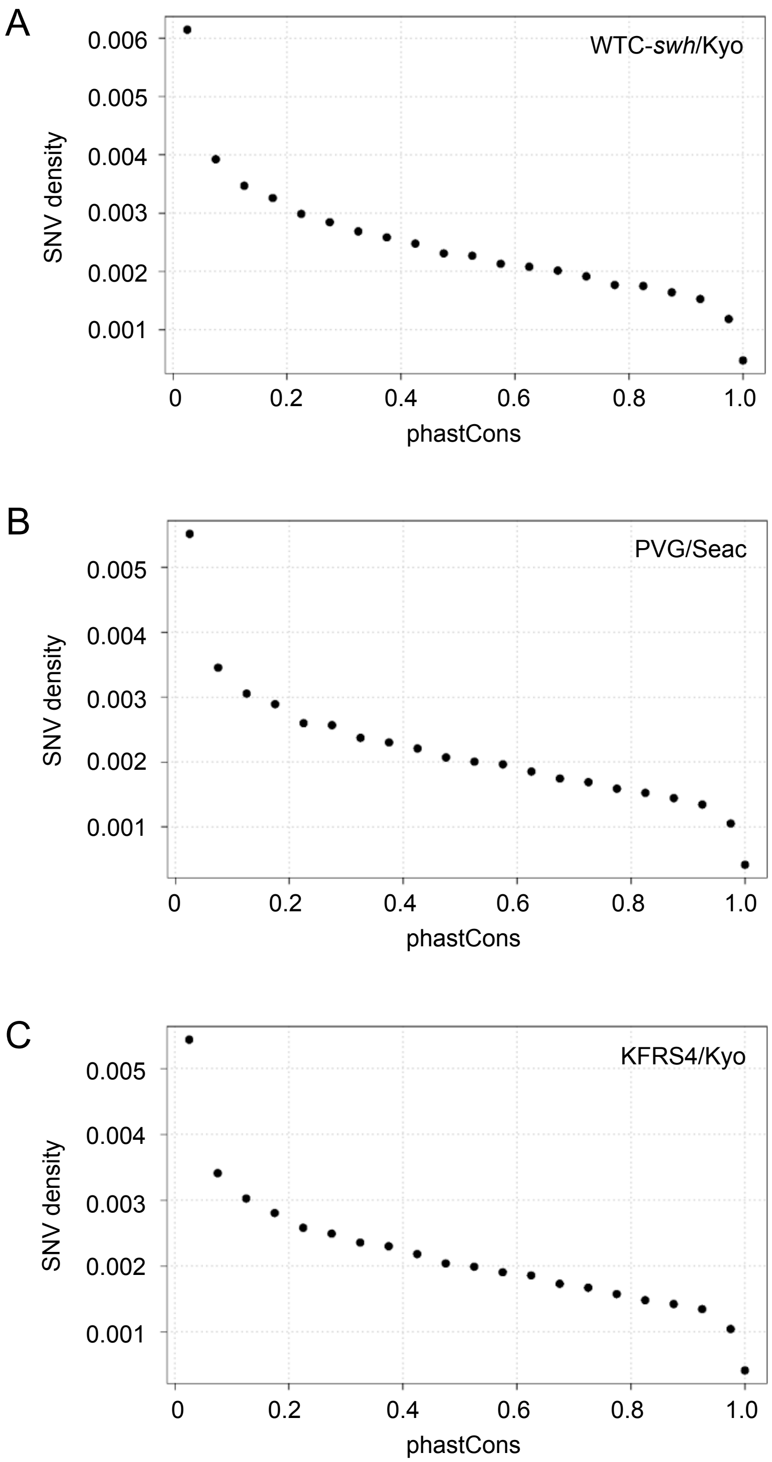

Figure S4. The relationship between the phastCons conservation score and SNV density for each rat strain. SNV density is calculated by the number of SNVs divided by the length of regions with a certain phastCons score range (binned for every 0.05 interval). (A) WTC-*swh*/Kyo, (B) PVG/Seac, and (C) KFRS4/Kyo.
